# Adult stem cells and tissue engineering strategies for salivary gland regeneration: a review

**DOI:** 10.1186/2055-7124-18-9

**Published:** 2014-07-24

**Authors:** Chankee Yoo, Jeremy B Vines, Grant Alexander, Kyle Murdock, Patrick Hwang, Ho-Wook Jun

**Affiliations:** Department of Biomedical Engineering, University of Alabama at Birmingham, Shelby Building 806, 1825 University Boulevard, Birmingham, AL 35294 USA; Department of Otorhinolaryngology-Head and Neck Surgery, CHA Bundang Medical Center, CHA University, 59 Yatap-ro, Gyeonggi-do, Bundang-gu, Seongnam-si, 463-712 South Korea

**Keywords:** Hyposalivation, Mesenchymal stem cells, Tissue engineering, Salivary gland, Xerostomia

## Abstract

Saliva is an important compound produced by the salivary glands and performs numerous functions. Hyposalivation (dry mouth syndrome) is a deleterious condition often resulting from radiotherapy for patients with head and neck cancer, Sjogren’s Syndrome, or as a side effect of certain medications. Hyposalivation negatively affects speaking, mastication, and swallowing in afflicted patients, greatly reducing their quality of life. Current treatments for this pathology include modifying lifestyle, synthetic saliva supplementation, and the utilization of salivary gland stimulants and sialagogues. However, many of these treatments do not address the underlying issues and others are pervaded by numerous side effects. In order to address the shortcomings related to current treatment modalities, many groups have diverted their attention to utilizing tissue engineering and regenerative medicine approaches. Tissue engineering is defined as the application of life sciences and materials engineering toward the development of tissue substitutes that are capable of mimicking the structure and function of their natural analogues within the body. The general underlying strategy behind the development of tissue engineered organ substitutes is the utilization of a combination of cells, biomaterials, and biochemical cues intended to recreate the natural organ environment. The purpose of this review is to highlight current bioengineering approaches for salivary gland tissue engineering and the adult stem cell sources used for this purpose. Additionally, future considerations in regard to salivary gland tissue engineering strategies are discussed.

## Introduction

Saliva is a watery substance produced by the salivary glands. It consists of mucus, various electrolytes, glycoproteins, enzymes, and antibacterial compounds such as lysozyme and IgA [1]. In relation to its excretory composition, saliva performs many important functions such as breaking down starch into maltose for digestion, aiding in basic oral functions (e.g. speaking, mastication, and swallowing), and protecting against microbial related pathologies such as dental caries and periodontitis. Thus, any dysfunction or cessation to saliva production is a significant clinical concern. Hyposalivation, which is a characteristic of xerostomia (dry mouth syndrome), can significantly reduce the quality of life for afflicted patients [2, 3]. Major causes of hyposalivation include Sjögren’s Syndrome (SS) [4], γ-irradiation therapy for patients with head and neck cancer [5], and results of side effects from various medications and ectodermal dysplasias [6–8].

Current treatments for hyposalivation include symptom management, lifestyle changes, synthetic saliva supplementation, and the utilization of salivary gland stimulants and sialagogues (e.g., the muscarinic receptor agonists pilocarpine and cevimeline) which stimulate salivary secretion from residual acinar cells [9, 10]. Salivary gland stimulation via supplementation of cytokines or small molecules such as pilocarpine or amifostine to increase the secretion of saliva is the most commonly used treatment. However, this only treats surface level symptoms, provides temporary relief, and is plagued by multiple multiple side effects such as excessive sweating, chills, dizziness, excessive tearing, flushing, voice change, stuffy nose, tremor, nervousness, and diarrhea [11, 12].

Thus, development of an alternative treatment to provide a long-term and effective solution solution is imperative [13]. In order to address the limits associated with current therapies, many research groups have investigated various regenerative medicine strategies utilizing different stem cell sources to engineer artificial salivary tissues that can mitigate the effects of xerostomia and hyposalivation.

Tissue engineering is defined as the application of life sciences and materials engineering toward the development of tissue substitutes that are capable of mimicking the structure and function of their natural analogues within the body [14]. The general underlying strategy behind the development of tissue engineered organ substitutes is the utilization of a combination of cells, biomaterials, and biochemical cues intended to recreate the natural organ environment. This article is intended to provide a review of the most current approaches to utilizing stem cells and bioengineering principles for the purpose of salivary tissue regeneration.

## Review

### Anatomy of the salivary glands

Within the body, there are three main sources of saliva production: The parotid gland, the submandibular gland, and the sublingual gland. Additionally, there are 800–100 minor salivary glands throughout the oral cavity that produce a small amount of saliva, although, this amount is considerably less than the three major salivary organs [15].

Each of the salivary glands is located in various locations throughout the oral cavity in humans [16]. The parotid glands are the largest of the salivary glands and are located at the back of the mouth adjacent to the mandibular ramus. The submandibular glands are a pair of glands located superior to the diagastric muscles and below the jaws, juxtaposed and perpendicular to the mandibular ramus. The saliva produced by the submandibular glands accounts for roughly 70% of the saliva present within the oral cavity, despite the fact that they are smaller than the parotid glands. The sublingual glands are a pair of glands located anterior to the submandibular glands and inferior to the tongue and are responsible for the production of around 5% of the saliva present within the oral cavity. Each of the glands is encapsulated within connective tissue and arranged into lobules.

In terms of their growth and development, each of these organs are formed from two different layers of the three primary embryonic germ layers [17]. The submandibular and sublingual glands are derived from the endoderm, whereas the parotid gland is of ectodermal origin. The parotid gland produces a serous, watery secretion while the submandibular and sublingual glands produce secretions that contain mucus as well [16].

**Figure 1:**
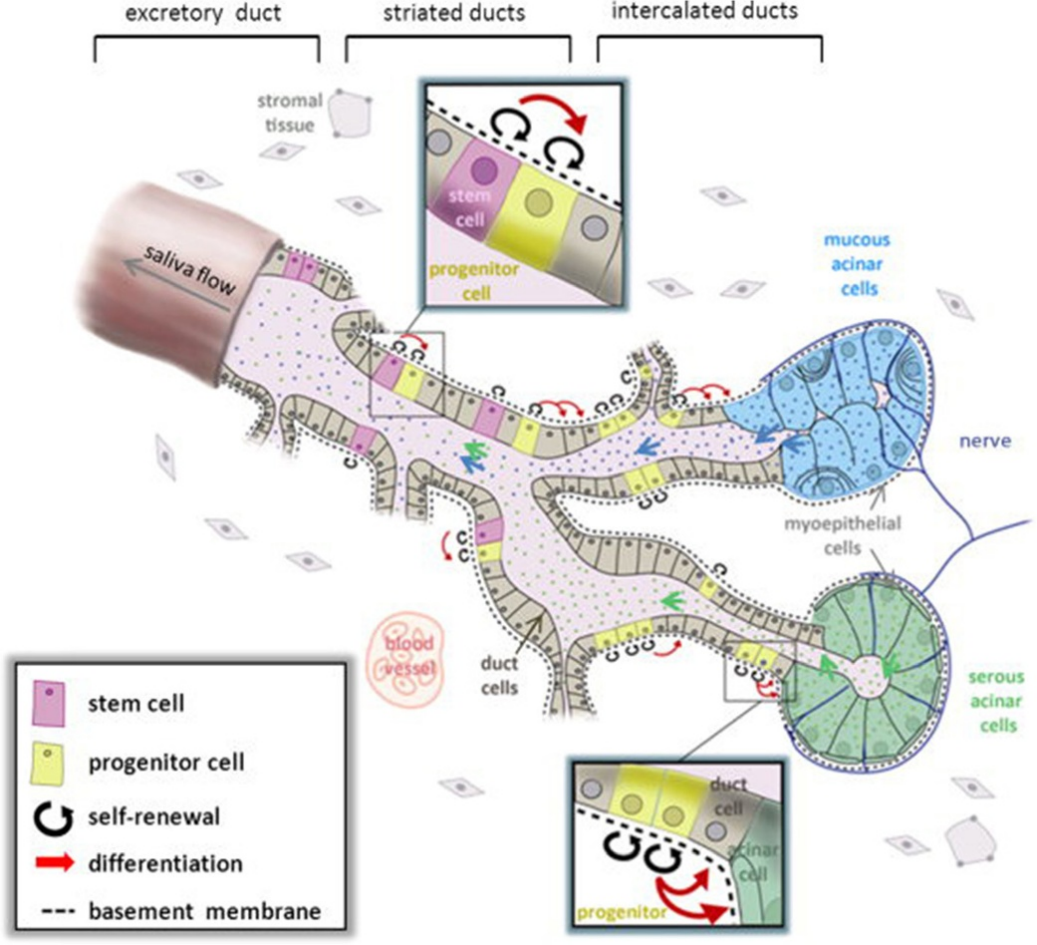
**Schematic representation of a generic salivary gland showing component cell types and theorized stem and progenitor cell locations.** Reproduced with permission from: [18].

The salivary glands of most mammals are made up of three main cell types: serous producing acinar cells, mucous producing acinar cells, and myoepithelial cells [16]. Serous producing acinar cells have a pyramidal morphology and are joined together to form spheroidal shapes while mucous producing acinar cells are cuboidal in shape and group together to form tubules. Myoepithelial cells are located near the ductal openings and serve to contract the ducts in order to squeeze out salivary secretions (Figure 1) [18].

The effects of radiotherapy on salivary gland cells are confounding. Theoretically, saliva producing acinar cells are not predicted to be radiation sensitive due to the fact that they are post-mitotic in nature [19]. However, despite this, irradiated salivary glands exhibit severe, early losses in saliva production [20]. In this regard, it is disputed as to whether radiation induced hyposalivation is a result of apoptosis or dysfunction resulting from radiation induced damage to the membranes of acinar cells [18, 21–24]. During the later phases of radiotherapy induced hyposalivation, functionally mature acinar cells cease their proliferation and are not replaced. It is suggested that the population responsible for replacing mature acinar cells, known as salivary gland progenitor cells (SSPCs), lose their regenerative capability as a result of radiation induced damage as well [20, 25, 26]. Due to this fact, much effort has been expended towards assessing various stem cell sources and materials to serve as potential replacements for SSPCs damaged during radiotherapy.

The purpose of this review is to highlight current bioengineering approaches for salivary gland tissue engineering and the adult stem cell sources used for this purpose. Some of the most currently studied adult derived stem cell populations for the purpose of salivary gland regeneration include salivary gland-derived stem cells (SGSCs), mesenchymal stem cells (MSCs), and human amnion epithelial stem cells (hAECs).

### Salivary gland-derived stem cells

Since adult stem cells are generally restricted to cell lineages of the body part of origin, researchers have attempted to use stem cells derived from salivary glands (SGSCs) to reduce hyposalivation and restore natural function [17]. Thus far, several approaches have been taken towards isolating and characterizing these stem cells. Some groups have sequestered cells from the parotid gland [27], the submandibular gland [28], a combination of both glands [29], and by co-culture [30].

Stem cells from the human parotid gland, removed via lateral parotidectomy, have been isolated and characterized *in vitro* [27]. Flow cytometry analysis showed that these stem cells were strongly positive for classic MSC markers (CD13, CD29, CD44, and CD90) and negative for key hematopoietic stem cell (HSC) markers (CD34, CD45). These SG-derived stem cells further displayed MSC-like characteristics by demonstrating the ability for adipogenic, osteogenic, and chondrogenic differentiation when grown in their respective induction media.

Stem cells isolated from a combination of the human parotid and submandibular glands were revealed to have a certain capacity for *in vivo* recovery of salivary gland function in radiation-damaged rat salivary glands [29]. Prior *in vitro* experiments confirmed that the SGSCs expressed MSC markers (CD44, CD49f, CD90, and CD105), excluded HSC markers (CD34, CD45), differentiated into MSC lineages, and could differentiate into amylase-expressing cells. Radiation-induced hyposalivation in rats was generated using an x-ray irradiator and human SGSCs (hSGSCs) were transplanted into the glands. After 60 days, the average saliva flow rate of the irradiation-damaged, hSGSC-treated group was twice that of the PBS-treated, irradiation-damaged group but was still lower than the undamaged group. Treatment with hSGSC was also quantified by measuring the rat body weight over time; the average body weight of hSGSC-treated rats was slightly increased in comparison to the PBS-treated rats.

**Figure 2:**
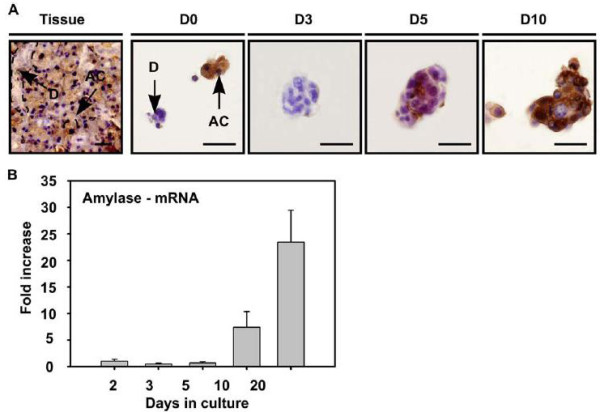
**Differentiation of salisphere into acinar cells. (A)** Amylase expressing cells (AC) in submandibular gland tissue (Tissue) were also present at the onset of culture (A-D0), and were visualized in the sphere at the onset of day 5 (A-D5), whereas granulae-containing spheres appeared in culture at later time-points (A-D10). Antibody labeling is shown in brown, nuclei in blue. Scale bar = 50 mm. (D = duct cells, AC = acinar cells, D0–3– 5–10 represent days in culture). **(B)** Real time RT-PCR confirmed the enhanced expression of amylase during in vitro culturing and differentiation. Error bars represent SEM (N = 2). Amylase mRNA expression levels at 2 days of culture were normalized to one. Reproduced with permission from: [28].

By using a floating sphere culture, further *in vitro* characterization of submandibular-derived SGSCs revealed cellular expression of Sca-1, c-Kit, and Musahsi-1 [28]. Immunohistochemical staining over a 10 day period was performed to analyze the origination and development of cell spheres. Initial H&E, Periodic Acid Schiff (PAS), CK7, and CK14 staining showed that cultured spheres contained acinar and ductal cells. Interestingly, acinar cells mostly disappeared by the third day but reappeared within the existing ductal spheres by the fifth day in culture. By day ten, acinar cells dominated sphere composition and amylase expression, quantified using RT-PCR, increased almost 25-fold after 20 days (Figure 2).

The *in vitro* results suggest that these sphere-forming cells originate from salivary gland ducts and are able to differentiate into amylase-producing, acinar-like cells. To analyze the stem cell characteristics of these spheres (now termed salispheres), common stem cell markers (Sca-1, c-Kit, and Musashi-1) were fluorescently labeled and visualized in culture. Sca-1 and c-Kit expression was seen in excretory duct cells but not acinar cells. Results from the H&E/PAS staining were confirmed by the fluorescent microscopy, which indicated a peak Sca-1 expression at 5 days.

Intraglandular transplantation of 3-day cultured salispheres into irradiated mice resulted in the formation of ductal structures near the injection site. There was an increase in acinar cell surface area in salisphere-treated mice compared to the untreated group. Ninety days after irradiation, saliva production in salisphere-treated mice was higher than the untreated counterparts and correlated strongly with acinar surface area. After purifying salispheres to a c-Kit^+^ population, cells were capable of differentiating into acinar cells *in vitro* and transplantation of a small number of cells (300–1000 per gland) improved saliva production in 69% of irradiated mice *in vivo*, after 120 days.

However, a major limitation for SGSC therapy in the treatment of radiation-induced hyposalivation is the difficulty with isolating autologous stem cells from a severely injured gland. To overcome this barrier, a co-culture system of mouse embryonic stem cells (mESCs) and human SG fibroblasts was developed to facilitate differentiation of mESCs to SGSCs [30]. After 1 week in co-culture, a significant change in cell morphology was found and RT-PCR results showed a sudden appearance of amylase and bFGF. These GFP-expressing SG cells were transplanted into normal mice submandibular glands and histology was performed after 1 month. H&E and PAS staining of SGSC-treated mice showed normal formation of ductal and acinar structures. Fluorescent microscopy of the GFP-positive donor cells qualitatively confirmed the cells’ ability to integrate into the existing tissue. Even though this method is not confined by the need for autologous stem cells from a radiation-damaged gland, it is limited by the ethical concerns surrounding embryonic stem cells and their lack of availability in clinical settings.

### Bone marrow mesenchymal stem cells (MSCs)

Mesenchymal stem cells (MSCs) are multipotent stem cells capable of differentiating into many cell types, including chondrocytes, adipocytes, osteoblasts, acinar cells, and salivary epithelial cells [31–34]. Their potential to repair damaged tissues, anti-inflammatory effects, and low immunogenicity make MSCs strong candidates for both experimental investigations *in vitro* and *in vivo* as well as clinical treatment of various diseases [31–35]. Therefore, MSCs were investigated for regeneration and functional restoration of the salivary gland.

### MSC implantation and Sjögren’s syndrome

**Figure 3:**
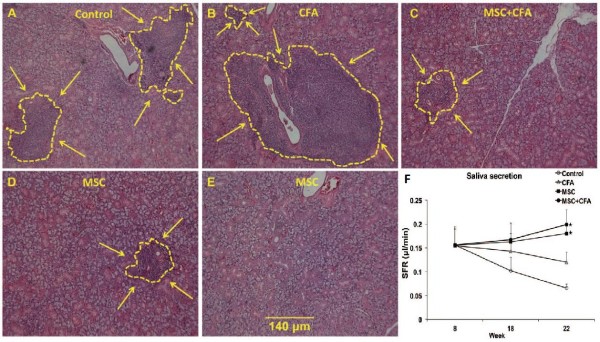
**Lymphocytic infiltrates in salivary glands of NOD mice.** A–E: H&E staining showing lymphocytic infiltration size (shown by yellow line and arrows) in NOD that were: untreated **(A)**, CFA-treated **(B)**, MSC + CFA **(C)**, MSC **(D)**. In 2 of the 5 NOD mice transplanted with MSCs only, no lymphocytic infiltrates were noted **(E)**. Scale bar: 140 um for all images. **(F)**: Salivary flow rates (SFRs) of NOD mice. SFRs in MSC + CFA (black circle; n = 10) and MSC (black square; n = 5) groups did not decrease during the follow-up period (22 wk of age) and were significantly higher than SFRs of CFA-treated or control NOD groups (n = 5 per group; P < 0.05). SFRs in CFA (triangular) or control (untreated; open circle) groups continued to decrease during the follow-up period (*P < 0.05). Reproduced with permission from: [35].

Two studies investigated the role of MSCs as a therapeutic option for treatment of Sjögren’s syndrome (SS), a chronic autoimmune disorder that results in exocrine gland inflammation, impaired salivary function, and lymphocytic infiltrates within the salivary glands [31, 35]. Khalili et al. [35] used NOD mice with a Sjögren’s syndrome-like disease to investigate the effect of MSCs in reducing lymphocytic infiltrates in the salivary gland and restoring salivary function (Figure 3). They found that intravenous injection of MSCs reduced lymphocytic infiltrate and inflammation in the salivary gland compared to untreated controls, including a 10-fold decrease in the inflammatory cytokine TNF-α. MSC injection also preserved the saliva flow rate over the 14 week post-treatment period; moreover, when MSCs were administered in conjunction with complete Freund’s adjuvant (CFA), the salivary gland regenerative potential increased (Figure 3). These findings indicate that MSC therapy alone reduced inflammation, but there was additional tissue repair and regeneration when administered in conjunction with CFA.

Xu et al. [31] investigated the therapeutic effects of allogeneic bone marrow-derived MSCs in both preventative and therapeutic interventions using NOD/Ltj mice with Sjögren’s syndrome-like autoimmune disorders. As SS symptoms in mice typically manifest around 7-8 weeks of age, preventative infusions of MSCs were given at an age of 6 weeks while the treatment group received MSC infusions at 16 weeks of age. MSC infusion significantly decreased submandibular gland inflammation in both preventative and treatment groups by supporting Treg and Th2 differentiation while limiting Th17 and Tfh responses. Additionally, preventative infusions of MSCs resulted in sustained saliva flow rates; saliva flow rates significantly increased after 2 weeks in the MSC treatment group. These outcomes indicate that allogeneic MSCs were effective in both preventing and reducing inflammatory responses as well as sustaining and restoring salivary gland function in SS-like autoimmune mice [31]. Importantly, Xu et al. also conducted a clinical investigation in the efficacy of allogeneic MSC treatment in 24 human patients with primary SS, including 11 with xerostomia [31]. Allogeneic MSC infusions were tolerated well by all 24 patients, with no adverse events reported either during or post-MSC infusion. Furthermore, all patients displayed symptom improvements after MSC treatment, although the response time ranged from 2 weeks to 6 months. For the 11 patients with xerostomia, 2 weeks after MSC treatment the unstimulated salivary flow rate significantly increased; after 1 month, it exhibited a 2-fold increase, and continued to rise on follow-up visits. Stimulated salivary flow rate also significantly increased at follow-up over the course of 12 months. Overall, these findings demonstrate that the MSC treatment in human patients was well tolerated, inhibited the inflammatory response, significantly increased salivary flow rate, and improved SS disease symptoms, indicating that allogeneic MSC treatment is a safe and effective therapy for patients with SS and xerostomia.

### MSC therapy for radiation damaged salivary gland regeneration

Several groups explored the effects of MSCs on radiation-induced damage to salivary glands [32–34]. Sumita et al. [32] investigated the capacity of intravenously injected MSCs to differentiate into salivary epithelial cells and restore function to the salivary gland of mice exposed to head and neck irradiation. Salivary flow rate significantly increased at 8 and 24 weeks post-radiation in MSC-treated mice compared with untreated controls: at 8 weeks it was 2-fold higher, and had increased to a level comparable to that of normal mice. Compared to untreated controls, MSC-treated mice displayed significantly reduced cell apoptosis, a 2.5-fold increase in blood vessel percent, a significantly increased number of proliferative salivary epithelial cells, and significantly higher regeneration of acinar cells [32]. Moreover, transplanted MSC differentiation into salivary epithelial cells was observed. These results indicate that MSCs have vasculogeneic and paracrine effects that increase acinar cell proliferation and inhibit cell apoptosis, as well as the capacity to directly differentiate into salivary epithelial cells. Thus, MSCs can restore gland function and regenerate radiation-damaged salivary tissue.

Lin et al. [33] studied the therapeutic potential of MSCs for salivary gland regeneration both *in vitro* and *in vivo*. After 3 weeks of co-culture (MSCs and acinar cells), about half of the MSCs had differentiated into acinar-like cells, demonstrating MSC differentiation capacity *in vitro*. Both MSCs and differentiated acinar-like cells significantly increased saliva production, salivary gland weight, and body weight when transplanted into radiation-treated mice; these systemic and local effects indicate salivary gland regeneration. Moreover, after 43 days, transplanted MSCs were found to be integrated into the salivary gland and transdifferentiated into acinar-like cells. Therefore, transplantation of either MSCs or differentiated acinar-like cells may aid regeneration and restore functional salivary glands.

Lim et al. [34] investigated the effects of direct transplantation of highly homogeneous MSCs on salivary gland regeneration and functional restoration in mice after neck radiation. Irradiated mice that received MSCs showed significant increase in saliva flow rate and improvement in salivary gland weight compared to irradiated control mice that only received a PBS injection; moreover, the MSC treatment group had fewer apoptotic cells, higher numbers of functional acinar cells, and an increase in blood microvessel density. These results indicate that transplanted MSCs are capable of grafting into radiation-damaged salivary glands and preserving salivary gland function while reducing apoptosis and increasing microvessel density.

### Adipose-derived MSC therapy for radiation damaged salivary gland regeneration

Two studies explored the use of adipose-derived MSCs (AdMSCs) for salivary gland regeneration as these cells are readily available and are known to contribute to angiogenesis and to secrete multiple cytokines and growth factors [36, 37]. Kojima et al. [36] employed radiation to induce hyposalivation in mice in order to probe the regenerative potential of adipose-derived stromal cells (ADSCs) to restore salivary gland function. After percutaneous administration of ADSCs to the submandibular glands of irradiated mice, they found that saliva flow rate was significantly improved, recovering to about 75% of that found in normal mice after 5 weeks, while mice in the sham treatment group remained hyposalivary. The ADSC treatment group also tended to have more acinar cells, blood endothelial cell recovery to levels comparable to those of normal mice, and alleviation of the severe inflammatory infiltration found in the sham group. Furthermore, ADSC treatment displayed significant increases in angiogenesis enzymes and growth factors critical to salivary gland regeneration. These findings indicate that ADSC treatment can restore salivary gland function after radiation damage through paracrine effects, restoration of blood flow, and differentiation of ADSCs into endothelial cells. However, this study is limited by the fact that no ADSCs were observed to differentiate into acinar cells which affect hyposalivation directly.

Lim et al. [37] investigated the effects of multiple infusions of human adipose-derived MSCs (hAdMSCs) on salivary gland function in radiation damaged mice. 6 hours after the dose of radiation, mice were intravenously infused with hAdMSCs; infusions were performed again once a week for 3 consecutive weeks thereafter. At 12 weeks post-radiation, treated mice showed less periductal and perivascular fibrosis, significantly reduced numbers of apoptotic cells, and greater numbers of acinar cells compared to the untreated group. Differentiation of hAdMSCs into salivary gland cells was observed after 4 weeks *in vivo* as well as after co-culture *in vitro* with salivary gland cells. Treatment with hAdMSCs also significantly increased post-stimulation salivary flow rate compared to untreated controls, promoted regeneration of salivary gland cells, and provided protection against radiation damage to cells. These results show that xenogeneic hAdMSCs can migrate through the bloodstream to radiation-damaged salivary glands and promote functional recovery, indicating that hAdMSCs have potential for salivary gland restoration.

### Human amniotic epithelial cells

Recently, much attention has been dedicated towards the study of stem cells derived from placental tissues. Many studies have reported the isolation and identification of various pluripotent and broadly multipotent cell types from umbilical cord blood, amniotic and chorionic membranes, wharton’s jelly, and amniotic fluid [38–43]. In regard to the use of placental derived stem cells for salivary gland regeneration, only two studies have been done. In both studies, human amniotic epithelial cells (hAECs) were isolated and utilized for salivary gland acinar cell regeneration [44, 45].

hAECs are typically derived from the top-most layer of the amniotic membrane via trypsinization of the membrane following its collection during cesarean section. Human amniotic epithelial cells are similar to epithelial cells in the sense that they express common epithelial markers such as cytokeratin 7 (CK7) and are negative for CD44. However, unlike adult epithelial cells, these cells have been demonstrated to express characteristic markers of pluripotent stem cells such as stage specific embryonic antigen-4 (SSEA4), octamer binding protein-4 (oct-4), and Nanog [46]. Additionally, these cells have a stem-cell like character and demonstrate the capability to differentiate into a multitude of different lineages from all three embryonic germ layers such as osteocytes, adipocytes, neurons, hepatocytes, cardiomyocytes, and pancreatic cells [47, 48]. In the following studies, hAECs were differentiated into functional acinar cells by utilizing different methods.

In the first study, hAECs were isolated and co-cultured with submandibular salivary gland acinar cells of sqrague dawley rats using a double-chamber system for 1–2 weeks to induce their differentiation into salivary gland acinar cells [44]. At each time point, cells were analyzed with immunohistochemistry and RT-PCR for a variety of human and salivary gland specific factors. At the initial time point, hAECs were weakly positive for alpha amylase, however, expression increased 3.3 fold after 1 week and 6.6 fold after 2 weeks of co-culture with rat salivary gland acinar cells. Additionally, immunofluorescent staining confirmed cytokeratin 19 (CK19) expression in hAECs after 2 weeks. Both the immunofluorescent and RT-PCR analyses confirmed the capability of hAECs to trans-differentiate into salivary gland acinar cells.

In a separate study, isolated hAECs were injected into the irradiated glands of mice [45]. The glands were analyzed after 14 and 30 days using H&E and immunofluorescent staining. H&E staining revealed that irradiated glands treated with hAEC injections more closely resembled the histological structure of the non-irradiated controls. Immunofluorescent staining confirmed the expression of MAB1281 as well as CK7, cytokeratin 14 (CK14), and amylase. The presence of MAB1281 and CK7/CK14 after 30 days demonstrated that the salivary glands were still inhabited by human cells. These cells expressed salivary gland acinar cell specific markers indicate that they had trans-differentiated into saliva producing cells. Additionally, the salivary flow rate was also assessed. For irradiated salivary glands treated with hAEC injection, salivary flow rate at 30 days was restored to 48% of the non-irradiated controls. Overall, the study determined that intra-glandularly injected hAECs were capable of differentiating into acinar cells and restoring saliva production in irradiated mice, highlighting the potential of hAECs to serve as a stem cell source for salivary gland regeneration in clinical applications.

### Recent bioengineering approaches

One of the most common treatments for hyposalivation is oral administration of drugs for the stimulation of saliva flow. Muscarinic receptor agonists, such as pilocarpine and cevimeline, have been widely used as orally administered drugs for hyposalivation treatment [49–51]. However, this oral administration may cause a variety of side effects including nausea, diarrhea, dyspepsia, abdominal pain, dizziness, rhinitis, and hypertension [52]. The side effects may lead some patients to become uncomfortable with therapy and to return to palliative care. Thus, a controlled release of drugs at the salivary gland was considered to reduce the drug dosage which attenuates the occurrence of side effects [53]. Controlled drug release systems have been developed by utilizing various polymers such as hydrogels, [54] polymer based microchips, [55, 56] nanoshells, [57, 58] and microfluidics; [58] these systems enable drug supply to the target area with a desired release pattern. Commercial polymer hydrogels for a controlled release of pilocarpine have already been clinically tested in patients with Sjögren’s syndrome [53]. The pilocarpine-containing polymer hydrogel was placed into the buccal sulcus of the patients and it released in excess of 85% of loaded pilocarpine over 3 hours. Saliva and tear production were generally increased, and oral and ocular comfort scores assessed by visual linear analogue scale were also generally improved [53]. Poly(lactic-co-glycolic acid) (PLGA) microparticles were also developed for the controlled release of drugs in the salivary gland and evaluated for biocompatibility with the parotid tissue [59]. These controlled drug delivery systems potentially provide better management of salivary gland hyposalivation while having less adverse drug effects. However, the consistency of drug release kinetics, specific targeting, and the design and shape of drug carrier should be further verified for the effective treatment of hyposalivation. In addition, the severity of salivary hypofunction may be varied between patients, and patients with the most advanced stage may have little salivary tissue left [53]. Thus, the extent of salivary gland damage in each patient should also be carefully considered to determine the most effective drug therapy.

Gene delivery approach has also been considered as a potential therapeutic treatment for hyposalivation. Salivary glands have several advantages for clinical gene delivery [60]. Salivary glands are easily accessible for the treatment by gene-delivering vectors in a less-invasive manner. In addition, the gene-delivering vectors are well-encapsulated in the human salivary gland, which restricts the spread of vectors from the salivary gland [60]. We can also easily assess important physiological processes of salivary gland tissue, and it is not for life-threatening if severe unwanted complication occurs. General gene delivery techniques to major salivary glands are based on cannulation of parotid or submandibular ducts, which does not require local anesthesia and are readily injectable in the mouth [60]. Gene transfer into cells can be achieved using viral and non-viral vectors. Viral vectors are currently the most efficient vectors for gene transfer, but there are some safety concerns when using viral vectors; the risk of generating insertional mutagenesis, replication-competent virus, and immune responses may limit the clinical use of viral vectors [61–63]. On the contrary, non-viral vectors have less safety issues, but they show inefficiency of gene transfer in mammalian cells. For salivary glands, non-viral vectors are rarely used, whereas adenovirus type 5 (Ad5) and adeno-associated virus type 2 (AAV2) vectors are most often applied [60]. Ad5 vectors efficiently transduce salivary gland epithelial cells in various animals, such as mice, rats, and non-human primates, and generate the expression of the delivered gene in high levels, although they are transient due to a considerable immune response [64]. In contrast, AAV2-delivered gene expression remains much longer because they generate less host immune response, thus AAV2 vectors can be useful for studies requiring long-term expression [65]. However, AAV2 vector construction is more difficult than Ad5 vector creation, so we need to further understand its biology for convenient application [60]. Various salivary gene delivery applications for hyposalivation treatment have been reported; their applications in animal models demonstrated the great potential for hyposalivation treatment. Human aquaporin-1 (hAQP1) gene was transferred to pigs and rats for repair of irradiated salivary glands [66, 67]. In addition, manganese superoxide dismutase-plasmid/liposome (MnSOD-PL), basic fibroblast growth factor (bFGF), and vascular endothelial growth factor (VEGF) genes were transferred to mice for the prevention of radiation damage of salivary glands [68, 69]. For the treatment of Sjögren’s syndrome, Interleukin-10 (IL-10), interleukin-17 (IL-17), and vasoactive intestinal peptide (VIP) genes were also transferred to mice [70–72]. Among various delivery genes, hAQP1 gene encodes a water channel membrane protein which stimulates rapid water movement in response to an osmotic gradient. Thus, transferring hAQP1 gene to duct cells in radiation damaged salivary glands is expected to induce fluid secretion by providing stimulated water permeability pathways in duct cells [60, 66, 73]. A human clinical trial of hAQP1 gene delivery to the parotid glands of patients with radiation induced hyposalivation is ongoing [73, 74]. In this study, it was confirmed that the spread of treated Ad5-hAQP1 vector was limited by the gland capsule [74]. The patients apparently had a latent Ad5 infection in the targeted parotid gland which was activated after hAQP1 gene delivery. However, no virus or vector was detected in the patients’ serum [74]. Based on previous studies, gene delivery approaches can provide valuable translational possibilities for hyposalivation treatment. In order to provide better clinical availability, the long-term safety of gene delivering vectors and appropriate delivery genes should be further identified.

Recently, bone marrow-derived cells (BMC) mobilization by cytokine stimulation has also been reported for hyposalivation treatment [75, 76]. The subcutaneous injection of granulocyte colony stimulating factor (G-CSF) mobilized BMCs to the blood stream, which resulted in BMC migration to irradiated mouse salivary glands leading to improved morphology and function [75]. It was suggested that the BMC-mediated paracrine stimulation could enhance the glandular regeneration process. In addition, the combination treatment of G-CSF, FMS-like tyrosine kinase-3 ligand (Flt-3 L), and stem cell factor (SCF) further increased the number of different types of mobilized BMC; this treatment not only reduced the radiation-induced hyposalivation but also ameliorated the submandibular vascular damage through BMC-derived neovascularization [76]. This approach suggests clinical applicability for the use of BMC mobilization to improve radiation-induced damage. To utilize this treatment most effectively, the molecular mechanisms behind the observed protection and long-term duration must be further explored.

**Figure 4:**
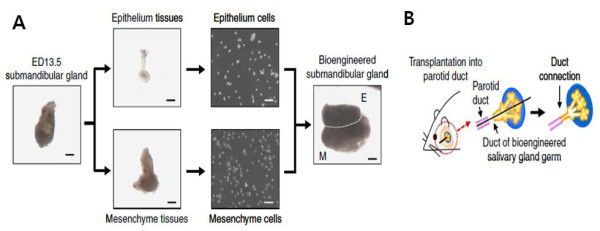
**Generation and transplantation of a bioengineered salivary gland germ. A**. Phase contrast images of the ED 13.5 submandibular gland germ, tissues, dissociated cells, and the bioengineered submandibular gland germ. E, epithelial cells; M mesenchymal cells. Scale bar, 200 μm. **B**. Schematic presentation of the transplantation procedure using the interepithelial tissue connecting plastic method with the bioengineered salivary gland germ. Reproduced with permission from: [78].

Various current approaches including the delivery of stem cell and therapeutic molecules such as drugs, genes, and cytokines, hold great promise to overcome the challenge of hyposalivation; however, they provide only partial restoration of the damaged salivary gland and its function. Thus, to achieve the complete functional replacement of lost or damaged tissue, a novel bioengineering approach reconstructing a fully functional organ was proposed [15, 77, 78]. This approach, called the “organ germ method”, demonstrated the regeneration of fully functional salivary glands in mice, which was induced by reciprocal epithelial and mesenchymal interactions through the engraftment of a bioengineered salivary gland germ. The bioengineered gland germs were constructed with epithelial and mesenchymal single cells obtained from each gland germ at mouse embryonic day 13.5-14.5; they then developed into a mature gland through acinar formations (Figure 4) [78]. The bioengineered submandibular gland showed the production of sufficient saliva in response to pilocarpine administration and gustatory stimulation by citrate and the recovery of swallowing in a salivary gland defective mouse model [78]. The organ germ method provides a proof-of-concept of regeneration by a bioengineered salivary gland as a potential treatment for hyposalivation. However, to realize the clinical practice of this method, the identification of an appropriate cell source for a bioengineered salivary gland germ should be established [15]. Thus, it will be necessary to identify somatic and other tissue-derived stem cell populations from the patient that have the capability to reproduce salivary gland organogenesis via epithelial-mesenchymal interactions.

## Conclusion

Many bioengineering and adult stem cell therapies for the treatment of hyposalivation are currently being developed and investigated. Most of these treatments rely on the utilization of allogeneic, adult derived stem cells. While many of these therapies are promising, there are still shortcomings that need to be addressed in the future. For example, the role that allogeneic stem cells play in regeneration has been shown to be transient in nature [79]. In this regard, it is suggested that allogeneic stem cells play two main roles in regeneration. First, allogeneic stem cells have immunosuppressive properties that allow them to quell inflammatory responses to encourage more robust healing. Secondly, they serve to recruit endogenous stem cell populations to the site of injury and encourage regeneration [80–82]. However, in an environment where no endogenous stem cell population is present (such as in the case of radiation induced xerostomia) and implanted stem cells are intended to serve as a functional replacement, this may be problematic. It has been reasonably determined that the short-lived nature of allogeneic stem cells is a by-product of the host immune response [83, 84]. Considering this fact, several strategies have been proposed in order to protect donor allogeneic cells from up-take by recipient immune responses.

The first strategy involves the use of immunoprotective biomaterials intended to isolate the donor stem cells from the recipient’s innate immune cells, yet, allow the diffusion of essential nutrients and oxygen necessary to maintain cellular viability. A few research groups have investigated the use of various biomaterials and configurations for this purpose [85–87]. A prominent example of this strategy in commercial development is the Encaptra system, which was developed for Viacyte’s allogeneic cellular therapy for the treatment of diabetes, PEC-01. This system consists of a pouch made up of three layers of polymeric meshing with decreasing porosity, with the innermost layer exhibiting the smallest pore size. The porosity of the outermost layers are amenable to the establishment of vascular networks, while the innermost layer prevents invasion by host immune cells, allowing the diffusion of oxygen and nutrients to the encapsulated therapeutic cell populations while isolating them from immunological attack. A second strategy, which may be used in conjunction with an immunoprotective barrier, is to induce the overexpression of various anti-inflammatory cytokines by therapeutic cell populations. In one such study, BMSCs were encouraged to overexpress the anti-inflammatory cytokine IL-10 using an mRNA transfection technology [88]. This serves to protect the allogeneic stem cells from the recipient’s immune response by suppressing the host’s immunological cell activity, thus allowing them to carry out their therapeutic function more efficiently.

An alternative solution to the problem of donor cell destruction is to utilize autologous cell populations capable of forming salivary gland acinar cells. In this sense, the ideal cellular source for this purpose is induced pluripotent stem cells (iPSCs). IPSCs, which are derived from adult somatic cells of the patient via reprogramming to express a subset of pluripotency genes and induce an embryonic-like state, would be an ideal candidate for autologous cellular therapy due to enhanced potency and immunological compatibility [89]. However, due to current safety concerns and associated regulatory barriers, the adoption of this technology is much more distant [90].

With regard to the differentiation of various stem cell populations, a majority of studies induce differentiation either by co-culture with isolated salivary gland cells or by injection into the salivary glands of mice. In the future, it would be beneficial to identify and utilize specific growth factors to create an induction media to ease the study of various stem cell types in-vitro and allow for consistency in deriving acinar-like salivary gland cells. Preliminary work has been performed to elucidate some of the conditions necessary to induce the differentiation of progenitor cell populations into salivary gland acinar-like cells. For example, one study found that TGF-b1 inhibited the expression of acinar cell associated genes from mesenchyme progenitors while TGF-bR1 inhibitor increased their expression [91]. Further work must be done in order to ascertain the specific growth factors necessary to create a standard induction media.

This paper is intended to provide an up to date review on the current status of salivary gland regeneration for the treatment of xerostomia induced hyposalivation. Overall, it has been demonstrated that tissue engineering approaches utilizing stem cells and biomaterials have the capability to become a viable means to achieve meaningful salivary gland regeneration. However, while preliminary in-vitro, animal, and human clinical studies have yielded promising results, it is clear that additional research is needed before salivary gland regeneration becomes a widespread therapeutic and clinical option.
